# Genome data of *Propionibacterium freudenreichii* J117, a functional strain from raw-milk cheese

**DOI:** 10.1016/j.dib.2026.112498

**Published:** 2026-01-24

**Authors:** Paulina Deptula, Jenni Sihvola, Pekka Varmanen

**Affiliations:** a Department of Food Science, University of Copenhagen, 1958 Frederiksberg, Denmark; b Department of Food and Nutrition, P.O. Box 66, University of Helsinki, FI-00014, Finland

**Keywords:** Food isolate, Genome sequence, Plasmid, PacBio, Vitamin B12

## Abstract

This dataset reports the complete genome sequence of *Propionibacterium freudenreichii* strain J117, a food-grade bacterium isolated from Austrian Vorarlberger Bergkäs cheese. The strain was selected for its application in a co-fermentation platform aimed at enhancing vitamin B12 content in plant-based fermented foods. Genomic DNA was extracted from anaerobic cultures grown in yeast extract lactate (YEL) broth and sequenced using PacBio Sequel II long-read technology with SMRT Cell 8 M. High-fidelity (HiFi) reads were generated, and circular consensus sequences (CCS) were assembled using the Improved Phased Assembler (IPA v2).

Genome annotation was performed with Bakta v1.10.4. Antibiotic resistance screening was carried out using the Resistance Gene Identifier (RGI v6.0.3) from the Comprehensive Antibiotic Resistance Database (CARD) via the PROKSEE platform. No plasmid-encoded resistance determinants were identified. The genome comprises two circular replicons and includes full annotation of coding sequences, RNAs, CRISPR array, and pseudogenes.

The raw sequencing data, genome assembly files, and annotation outputs are included in the associated data repository, organized in subfolders for raw reads, assemblies, and analysis results. This dataset supports the related research article: Zhang, R., Chen, L., Zhang, D., Sihvola, J., Chamlagain, B., Olin, M., Piironen, V., & Varmanen, P. Innovative co-fermentation of *Propionibacterium freudenreichii* and *Rhizopus oryzae* enhances vitamin B12, riboflavin, and flavor profile components in sweet fermented glutinous rice. *Food Chemistry*, 503 (2026).

The availability of this genome provides a reference for comparative genomic analysis, functional pathway prediction, and strain development. It also facilitates safety assessment of food-related strains, such as the absence of mobile antibiotic resistance genes, thereby supporting the transparent use of J117 in fermented food applications.

Specifications Table

**Table:** 

Subject	Biology
Specific subject area	Bacterial genomics; Fermentation;
Type of data	Genome assembly metrics, annotated gene data Raw Sequence Reads (FASTQ), Assembled (FASTA), Annotated (GenBank format).
Data collection	*Propionibacterium freudenreichii* J117 was isolated from Vorarlberger Bergkäse cheese (manufactured in Austria). Genomic DNA was extracted using the MagAttract HMW DNA Kit (Qiagen), quantified with NanoDrop and Qubit (Thermo Fisher). Sequencing was performed on a PacBio Sequel II platform (SMRT Cell 8 M, Binding Kit 2.0, Sequencing Kit 2.0) at the Institute of Biotechnology, University of Helsinki, Finland. Circular consensus reads were generated with SMRT Link v11 and assembled using IPA v2.
Data source location	University of Helsinki, Department of Food and Nutrition, Finland; (60.2267° N, 25.0152° E)
Data accessibility	Data is publicly available at the NCBI repository: BioProject accession: PRJNA1369162 BioSample accession: SAMN53383601, Genome accessions: CM132603 (chromosome); NZ_JBSNCU010000002 (plasmid) Direct URLs to data: https://www.ncbi.nlm.nih.gov/bioproject/PRJNA1369162/ https://www.ncbi.nlm.nih.gov/biosample/SAMN53383601/ https://www.ncbi.nlm.nih.gov/nuccore/NZ_CM132603.1 https://www.ncbi.nlm.nih.gov/nuccore/NZ_JBSNCU010000002.1 https://www.ncbi.nlm.nih.gov/datasets/genome/GCF_053839625.1/
Related research article	Zhang, R., Chen, L., Zhang, D., Sihvola, J., Chamlagain, B., Olin, M., Piironen, V. and Varmanen P. Innovative co-fermentation of *Propionibacterium freudenreichii* and *Rhizopus oryzae* enhances vitamin B12, riboflavin, and flavor profile components in sweet fermented glutinous rice. Food Chem. 503 (2026), doi:org/10.1016/j.foodchem.2025.147815.

## 1 Value of the Data

• The whole-genome sequence of *P. freudenreichii* J117 provides a new reference for a strain isolated from long-ripened raw milk cheese, supporting further strain-level comparative genomics within the species.
• These data enable deeper understanding of metabolic traits relevant to food fermentation, such as vitamin B12 biosynthesis, flavor compound production, and stress tolerance, thereby supporting research in functional food microbiology.
• The genome sequence may aid in identifying genetic determinants underlying enhanced sensory properties observed in co-fermentation applications with *Rhizopus oryzae*.
• The genome includes information relevant for strain safety evaluation, such as the absence of known virulence or transferrable antibiotic resistance genes, supporting its use in regulated food fermentation processes.
• This genome will be useful for future metabolic engineering or synthetic biology efforts aiming to optimize vitamin production or tailor aroma profiles in food-grade *P. freudenreichii* strains.

## 2 Background

*Propionibacterium freudenreichii* is a food-grade, vitamin B12-producing bacterium traditionally used in Swiss-type cheese production and increasingly investigated for fortifiying plant-based foods [1,2]. To support the development of novel co-fermentation strategies involving *P. freudenreichii* and *Rhizopus oryzae*, as reported in Zhang et al. [3], we isolated a new strain, *P. freudenreichii* J117, from Austrian raw-milk Bergkäs cheese. Whole-genome sequencing was undertaken to characterize the genetic features of J117, enable comparative analysis, and assess its suitability for food applications. Importantly, genomic characterization allows verification of food safety traits, including the absence of mobile antibiotic resistance determinants or virulence-associated elements, which is essential when deploying bacterial strains in fermented food production. The genome dataset provides a foundational resource for interpreting the nutritional and sensory outcomes reported in Zhang et al. [3] and supports future research on strain optimization and safe application of *P. freudenreichii* in fermentation processes.

## 3 Data Description

The dataset includes the complete genome sequence of *P. freudenreichii* strain J117, assembled from PacBio HiFi long reads generated on the Sequel II system using SMRT Cell 8 M chemistry. Assembly with IPA v2 resulted in two circular replicons: a 2.59 Mb chromosome with a GC content of 67.4 % and a 32.7 kb plasmid with 65.6 % GC, yielding a total genome size of 2.62 Mb at ∼100 × coverage.

Genome annotation identified 2280 coding sequences, comprising 2252 CDSs on the chromosome and 32 on the plasmid. Additional features included 45 tRNAs, 6 rRNAs, 1 tmRNA, 2 ncRNAs, 13 ncRNA regions, 1 CRISPR array, 14 pseudogenes, and a predicted origin of replication. The genome's overall coding density was 88.2 %.

Antimicrobial resistance screening detected two chromosomal hits: (i) *vanT* of the *vanG* operon, associated with low-level vancomycin resistance, but the remaining essential operon genes (*vanURSYWGXYT*) [4] were absent; and (ii) a point mutation in the 23S rRNA gene previously linked to macrolide resistance in *Cutibacterium acnes* [5,6]. No resistance determinants were detected on the plasmid.

## 4 Experimental Design, Materials and Methods

### 4.1 Bacterial isolation

A novel *P. freudenreichii* strain, designated J117, was isolated from artisan-style commercial Vorarlberger Bergkäse cheese (Austria; packaging date: 20 May 2021). The cheese was long-ripened, produced from unpasteurized cow milk and animal rennet, and contained 1.6 % NaCl and 30 % fat. The sample was purchased from a local grocery store in Finland. One gram of cheese was finely chopped and mixed with 10 mL of phosphate-buffered saline (PBS), followed by incubation at room temperature for 30 min with agitation (170 rpm). The mixture was serially diluted and plated onto yeast extract lactate (YEL) agar, composed of 10 *g*tryptone (Sigma-Aldrich), 10 *g*yeast extract (Becton Dickinson), 16.7 *g*of 60 % w/w DL-sodium lactate (Sigma-Aldrich), 2.5 *g*K₂HPO₄, 0.005 *g*MnSO₄, and 15 *g*Bacto-Agar (Sigma) per liter.

Plates were incubated anaerobically at 30 °C for 6 days using Anaerocult® A gas packs (Merck KGaA, Darmstadt, Germany). Colonies displaying typical *Propionibacterium* morphology were re-streaked and subjected to Gram staining and catalase testing. Colony PCR was then performed using two primer pairs targeting the *clpX* (RM25_0787) and *sdhA* (RM25_1351) genes in *P. freudenreichii* [7]: 5′-GACCTGTTCAAGTGCTCG-3′ / 5′-GCCACCTTGTCGATCTCG-3′,5′-GCCGGCGATGAGAAGTAC-3′ / 5′-CGTGGCGGCTGTGCATG-3′. PCR reactions were performed using Phusion Master Mix (Thermo Fisher Scientific, Waltham, MA, USA) supplemented with 3 % (v/v) DMSO. Amplicons were separated on 0.8 % (w/v) agarose gels (Bio-Rad) stained with SYBR™ Safe DNA Gel Stain; ThermoFisher Scientific) and visualized using an Alpha Imager HP system (ProteinSimple Inc., San Jose, CA, USA). One colony testing positive with both primer pairs was re-streaked on fresh YEL agar, designated as strain J117, and subjected to whole-genome sequencing.

### 4.2 Genomic DNA isolation

The culture was prepared from 15 % glycerol stock stored at −80 °C by streaking on a YEL agar plate and incubation at 30 °C in anaerobic jars (Anaerocult, Merck, Germany) for 4 days. For preparation of liquid culture, a single colony from the plate were picked and transferred to 15 mL Falcon tubes containing 10 mL of the liquid medium.

For DNA extraction, cells from 72 h liquid cultures were harvested by centrifugation at 2100 × *g* for 5 min at 4 °C and washed once with buffer (50 mM Tris, 10 mM EDTA, pH 8.0). Genomic DNA was extracted using the MagAttract HMW DNA Kit (Qiagen, Nordic). DNA quality and quantity were assessed using a NanoDrop spectrophotometer and a Qubit fluorometer (Thermo Fisher Scientific, USA), confirming sufficient yield for sequencing.

### 4.3 Genome sequencing, assembly and analysis

High-fidelity (HiFi) reads were generated on a PacBio Sequel II using SMRT Cell 8 M (S/P5-C2/5.0) with the Sequel II Binding Kit 2.0 (101–894–200) and Sequencing Kit 2.0 (101–826–100). Circular consensus sequences (CCS) were produced using SMRT Link v11 (ccs v6.3.0, basecaller v5.0.0) with the parameters –minPasses 3, –minSnr 2.5, and –minPredictedAccuracy 0.99 and assembled with PacBio’s Improved Phased Assembler (IPA) v2with resulting genome coverage of 100 ×.

Functional annotation was conducted using Bakta v1.10.4 [8]. Screening for antimicrobial resistance determinants was performed with the Comprehensive Antibiotic Resistance Database’s Resistance Gene Identifier (CARD RGI v6.0.3), accessed via the PROKSEE platform [9]. All tools were run with default settings.

## Limitations

Not Applicable.

## Ethics Statement

The authors have read and follow the ethical requirements for publication in Data in Brief and confirm that the current work does not involve human subjects, animal experiments, or any data collected from social media platforms.

## CRediT Author Statement

**Paulina Deptula:** Conceptualization, Methodology, Investigation, Writing- Original draft preparation **Jenni Sihvola:** Investigation, Writing- Original draft preparation **Pekka Varmanen:** Conceptualization, Supervision, Writing- Reviewing and Editing,

## Data Availability

NCBIGenome assembly ASM5383962v1 (Original data).
